# Influence of Root Post Materials and Aging on Fracture Strength and Marginal Gap Quality of Ceramic Crowns—An In Vitro Study

**DOI:** 10.3390/ma16113985

**Published:** 2023-05-26

**Authors:** Christoph-Ludwig Hennig, André Stöcker, Ann Nitzsche, Justus Marquetand, Collin Jacobs, Florentine Jahn

**Affiliations:** 1 Department of Orthodontics, Center of Dental Medicine, University Hospital Jena, An der alten Post 4, 07743 Jena, Germany; 2 Section of Geriodontics, Department of Conservative Dentistry and Periodontology, Center of Dental Medicine, University Hospital Jena, An der alten Post 4, 07743 Jena, Germany; 3 Department of Epileptology, Hertie-Institute for Clinical Brain Research, University of Tübingen, 72076 Tübingen, Germany; 4 Department of Neural Dynamics and Magnetoencephalography, Hertie-Institute for Clinical Brain Research, University of Tübingen, 72076 Tübingen, Germany; 5 MEG-Center, University of Tübingen, 72076 Tübingen, Germany

**Keywords:** root post material, marginal quality, artificial aging, loading capacity, titanium, glass fiber

## Abstract

The design of and materials for prosthodontic abutments and posts have significant influences on the fracture resistance of restored teeth. This in vitro study compared the fracture strength and marginal quality of full-ceramic crowns as a function of the inserted root posts via simulation of a five-year period of use. Test specimens were prepared from 60 extracted maxillary incisors using titanium L9 (A), glass-fiber L9 (B), and glass-fiber L6 (C) root posts. The circular marginal gap behavior, linear loading capacity, and material fatigue after artificial aging were investigated. The marginal gap behavior and material fatigue were analyzed using electron microscopy. The linear loading capacity of the specimens was investigated using the Zwick Z005 universal testing machine. None of the tested root post materials showed statistically significant differences in marginal width values (*p* = 0.921), except in the case of marginal gap location. For Group A, there was a statistically significant difference from the labial to the distal (*p* = 0.012), mesial (*p* = 0.000), and palatinal (*p* = 0.005). Similarly, Group B showed a statistically significant difference from the labial to the distal (*p* = 0.003), mesial (*p* = 0.000), and palatinal (*p* = 0.003). Group C showed a statistically significant difference from the labial to the distal (*p* = 0.001) and mesial (*p* = 0.009). Linear load capacity reached mean values of 455.8–537.7 N, and micro-cracks occurred after artificial aging, predominantly in Groups B and C. Through the chosen experimental design, it was shown that the root post material and root post length had no influence on the fracture strength of the test teeth before or after artificial aging. However, the marginal gap location depends on the root post material and its length, which is wider mesially and distally and also tends to be greater palatinally than labially.

## 1. Introduction

The restoration of endodontically treated teeth can be one of the most challenging tasks for the practicing dentist. A wide range of restorative materials, techniques, and systems are available. Both the longevity of the restoration and the possible future treatment needs are important considerations, especially when the patient is young. Furthermore, patient expectations regarding biocompatibility and aesthetics have increased in recent years [1,2]. Numerous studies have shown that the design and material of the post and abutment have significant influences on the fracture resistance of restored teeth [3,4,5,6,7]. However, there is no consensus in the literature regarding which technique and material are most suitable, as the obtained study data have shown a high degree of variation, partly due to the lack of generally applicable standards [6,7,8,9,10,11]. The situation regarding study of the clinical long-term behavior of metal-based root posts is better. The failure rates differ depending on the post shape, post length, and manufacturing method [12]. A wide variety of root post materials is available. In addition to aesthetic expectations, the root post material should meet the requirements of electrochemical safety, sufficient fracture strength, and high accuracy of fit with easy processing. These requirements are met by selected metals or metal alloys, high-strength ceramics, and fiber-reinforced composite materials [13]. In addition, properties such as rigidity and elasticity behavior are of increasing clinical importance [14]. Studies based on the finite element method have shown that biomechanical impairments occur when materials with a higher modulus of elasticity than dentin are used. In contrast, the use of materials with a modulus of elasticity comparable to that of dentin does not interfere with the stress profile within the root. Nevertheless, the material must be able to withstand high mechanical stresses [15]. Numerous studies of the modulus of elasticity of human dentin have yielded values of 10 to 30 GPa [16]. The modulus of elasticity of dental enamel is considerably higher—up to 87.5 GPa [17]. However, the influence of a low modulus of elasticity has been a subject of controversy and discussion in the literature. While some authors have advocated for root posts with dentin-like mechanical properties, others have emphasized the need for rigid root posts [18]. Despite the large number of studies, the available evidence is insufficient for making a clear recommendation regarding the material [19].

The material type and dimensions (length, diameter) of root post materials in terms of their loading capacity and marginal gap behavior were investigated in the present study. Another objective of this in vitro study was to compare the fracture strength and marginal quality of all-ceramic crowns as a function of the root posts used by simulating five years of aging. The study addresses the question of whether the head post design has a positive influence on fracture resistance and fatigue resistance.

## 2. Materials and Methods

### 2.1. Tooth Preparation

**Figure 1:**
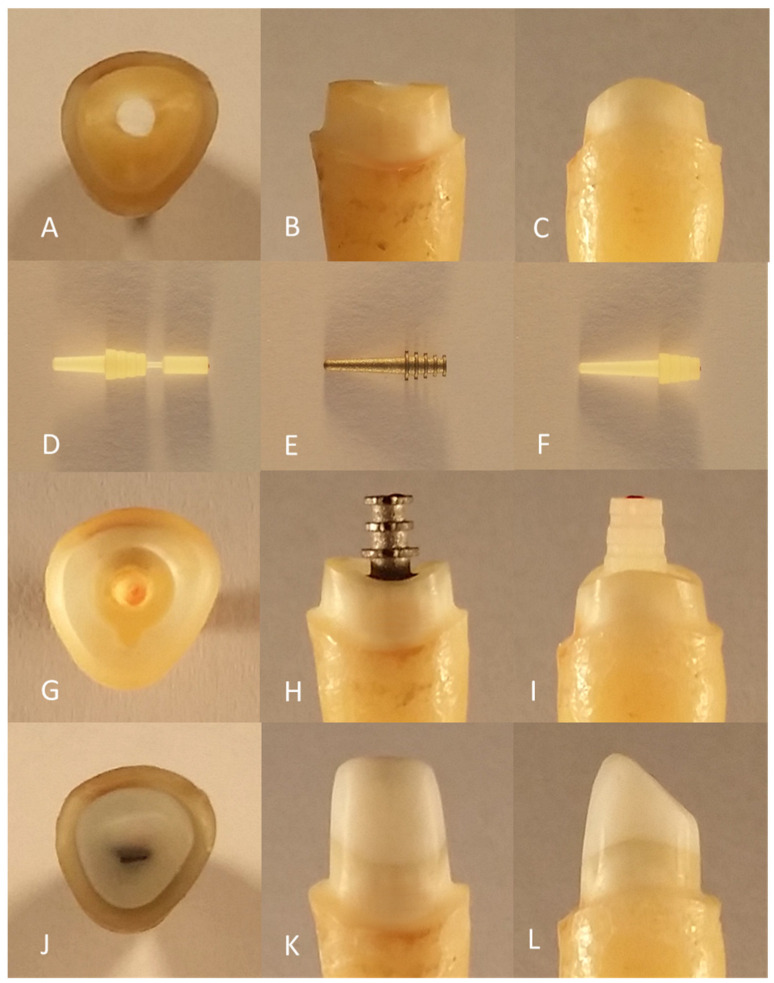
Preparation of the test specimen: pre-prepared tooth from incisal (**A**), vestibular (**B**), and distal viewpoints (**C**); root posts of glass-fiber L6 (**D**), titanium L9 (**E**), and glass-fiber L9 (**F**); post bed preparation of incisal (**G**), vestibular with titanium L9 (**H**) and distal with glass-fiber L9 (**I**); core build-up after fine preparation from incisal (**J**), vestibular (**K**), and distal viewpoints (**L**).

Initially, 95 human maxillary central incisors were identically pretreated. The teeth were stored in 0.1% thymol solution (Caesar & Loretz GmbH, Hilden, Germany) at room temperature and removed only during the working steps while taking care to maintain sufficient moisture. Beforehand, the teeth were cleaned using hand instruments (HS-Gracey curette Maxigrip 5/6, Henry Schein Dental Deutschland GmbH, Langen, Germany) and trephined with a spherical preparation diamond (Komet Gebr. Brasseler GmbH & Co. KG, Lemgo, Germany). The pulp was then removed, and root canal preparation was performed using K-drills and Hedström files (VDW GmbH, Munich, Germany) up to an ISO size of 50. The root canal preparation ended 0.5 mm before the anatomical apex. During and after preparation, the root canals were rinsed with 2% chlorhexidine digluconate solution (ParoEx, GUM, SUNSTAR Deutschland GmbH, Schönau, Germany) and dried with paper tips (Dentsply DeTrey GmbH, Konstanz, Germany). The root canal filling by lateral condensation was performed using ISO size 50 gutta-percha points (Coltène/Whaledent GmbH & Co. KG, Langenau, Germany) and AH Plus root canal filling paste (Dentsply DeTrey GmbH, Konstanz, Germany) using appropriate finger spreaders (VDW GmbH, München, Germany). The access cavity was provisionally sealed with Cavit (3M Deutschland GmbH, Neuss, Germany). The preparation was performed with the T1 LINE C 200 L high-speed contra-angle handpiece (Sirona Dental Systems GmbH, Bensheim, Germany) under constant water cooling. Using a cylindrical preparation diamond (Komet Gebr. Brasseler GmbH & Co. KG, Lemgo, Germany), the teeth were initially decapitated up to 2 mm coronal to the enamel–cement interface, leaving a circular coronal residual substance of 2 mm to guarantee an appropriate ferrule design. Using a preparation diamond with guide pin (Komet Gebr. Brasseler GmbH & Co. KG, Lemgo, Germany), a uniformly wide step of 0.59 mm was initially achieved (Figure 1A–C).

Following the preliminary preparation, the teeth were measured and examined for defects in an MBS-10 stereomicroscope (JSC ‘LZOS’—Lytkarino Optical Glass Factory, Lytkarino, Russia). The teeth that were used had a root length of at least 12 mm measured from the most apical point of the preparation margin to the apex. The mesial–distal width at the level of the preparation margin was at least 6 mm. In addition, a circular ferrule design of 2 mm was ensured. The pre-prepared teeth showed no residual caries and no restorations and had completed root growth. As far as could be seen, resorptions, infractions, and fractures were excluded. The requirements for use in the study were ultimately met by 60 teeth, which were distributed into six groups of 10 teeth each via computer-assisted randomization. The sample teeth in Group A (A0–A9) and Group D (D0–D9) were restored using titanium posts with a shaft length of 9 mm (titanium L9). The specimen teeth in Group B (B0–B9) and Group E (E0–E9) were supplied with glass-fiber posts with a shaft length of 9 mm (glass-fiber L9). The specimen teeth in Group C (C0–C9) and Group F (F0–F9) were supplied with glass-fiber posts with a shaft length of 6 mm (glass-fiber L6).

**Figure 2:**
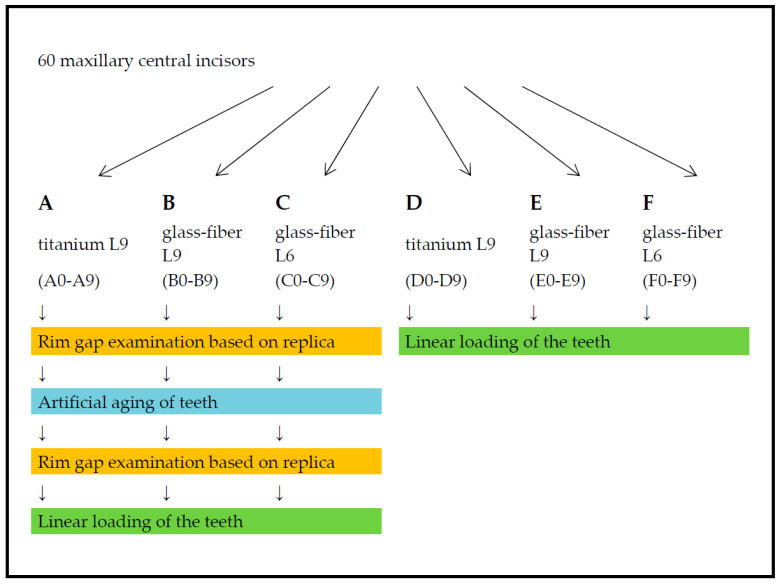
Flowchart of the experimental arrangement for the investigation.

The specimen teeth in Groups A, B, and C were artificially aged by thermocycling and mechanical loading (TCML) with preceding and subsequent marginal gap analysis. Finally, linear loading was performed to determine the fracture strength. For the specimen teeth in Groups D, E, and F, only linear loading was performed to determine the fracture strength. The test arrangement of the investigation is shown in Figure 2.

### 2.2. Production of the Test Specimens

All the posts used were acquired from ER system (Erlanger root post assembly system) from Komet (Komet Gebr. Brasseler GmbH & Co. KG, Lemgo, Germany) and have an equally dimensioned coronal retentive head. In addition, the titanium L9 (Komet Gebr. Brasseler GmbH & Co. KG, Lemgo, Germany) and glass-fiber L9 (Komet Gebr. Brasseler GmbH & Co. KG, Lemgo, Germany) posts have identical shaft dimensions. Accordingly, the post bed preparation of titanium L9 and glass-fiber L9 was methodologically the same. The glass-fiber L6 post (Komet Gebr. Brasseler GmbH & Co. KG, Lemgo, Germany) has a 3 mm shorter shaft with approximately the same cervical diameter and identical pitch angle. For better handling, the glass-fiber L6 post has a coronally seated handling part. Figure 1 shows the root posts used in this study (Figure 1D–F).

The post bed preparation was performed using the T1 LINE C 40 L transfer contra-angle handpiece (Sirona Dental Systems GmbH, Bensheim, Germany) under constant water cooling. Initially, the root filling was removed over a length of 11 mm from the reference point with a pilot drill (Komet Gebr. Brasseler GmbH & Co. KG, Lemgo, Germany) at a speed of 1200 rpm. The central inlay cavity with a depth of 2 mm from the reference point was prepared with a face grinder (Komet Gebr. Brasseler GmbH & Co. KG, Lemgo, Germany) at a speed of 2000 rpm. Subsequently, a rotation protection groove was prepared using the preparation diamond (Komet Gebr. Brasseler GmbH & Co. KG, Lemgo, Germany) at the widest point of the inlay cavity. To widen the root canal, the corresponding depth gauge (Komet Gebr. Brasseler GmbH & Co. KG, Lemgo, Germany) was placed on the enlarger (Komet Gebr. Brasseler GmbH & Co. KG, Lemgo, Germany). Using a speed of 1000 rpm, the channel was widened until the depth gauge was in contact with the central inlay cavity.

In the meantime, the canal was rinsed with 2% chlorhexidine digluconate solution (ParoEx, GUM, SUNSTAR Deutschland GmbH, Schönau, Germany). After the root canals were dried with paper tips, the accuracy of fit of the prepared post bed with the post was checked (Figure 1G–I). For mechanical conditioning of the canal wall, a roughening instrument (Komet Gebr. Brasseler GmbH & Co. KG, Lemgo, Germany) was manually rotated for three turns in the root canal without pressure. The glass-fiber L9 (Komet Gebr. Brasseler GmbH & Co. KG, Lemgo, Germany) and glass-fiber L6 (Komet Gebr. Brasseler GmbH & Co. KG, Lemgo, Germany) posts were methodically cemented with the same adhesive using DentinBuild Evo dual-curing, flowable radiopaque hybrid composite (Komet Gebr. Brasseler GmbH & Co. KG, Lemgo, Germany). The coronal build-up was performed in the sense of a monoblock with the same material. The titanium L9 post (Komet Gebr. Brasseler GmbH & Co. KG, Lemgo, Germany), on the other hand, was conventionally cemented with DentinBond Evo (Komet Gebr. Brasseler GmbH & Co. KG, Lemgo, Germany), followed by an equally adhesive coronal build-up with DentinBuild Evo30. Table 1 lists the manufacturer’s specifications for the root posts used.

Next, fine preparation was performed with the T1 LINE C 200 L high-speed contra-angle handpiece (Sirona Dental Systems GmbH, Bensheim, Germany) and the preparation diamond (Komet Gebr. Brasseler GmbH & Co. KG, Lemgo, Germany). An optimum speed of 20,000 rpm was used to aim for a convergence angle of 6°. A 1 mm-wide circular step with a rounded inner edge was created while retaining the pre-prepared restoration margin. The abutment was incisally shortened so that the die had a uniform length of 6 mm labially and 3 mm palatally (Figure 1J–L).

Finally, the prepared teeth were restored using all-ceramic crowns and the CEREC CAD/CAM system (Sirona Dental Systems GmbH, Bensheim, Germany). VITA-BLOCS Mark II monochromatic feldspat ceramic blanks (VITA Zahnfabrik H. Rauter GmbH & Co. KG, Bad Säckingen, Germany) were used. Digital impressions were taken with the CEREC Bluecam (Sirona Dental Systems GmbH, Bensheim, Germany). For this purpose, the sample teeth were positioned in a phantom jaw with a gingival mask (KaVo Dental GmbH, Biberach/Riß, Germany), considering tooth axis and rotation. Prior to scanning, the surface was coated evenly with a thin layer of Scandry scan spray (Dentaco GmbH & Co. KG, Essen, Germany). The crowns were modeled using CEREC SW 4 software (Sirona Dental Systems GmbH, Bensheim, Germany) with the Biogeneric individual design procedure and manual finishing to ensure a minimum layer thickness of 1 mm and an incisal offset of 3 mm. The fit of the crowns was checked with occlusion spray (Henry Schein Dental Deutschland GmbH, Langen, Germany) and a dental probe to ensure that there was a smooth transition between the tooth and the crown. Subsequently, the attachment point of the crown, which was always located labially, was ground with a diamond grinder (Komet Gebr. Brasseler GmbH & Co. KG, Lemgo, Germany). The specified minimum wall thickness and the incisal offset were checked again using calipers (Henry Schein Dental Deutschland GmbH, Langen, Germany). Finally, the respective specimen number was finely engraved on the labial surface using a spherical diamond bur (Komet Gebr. Brasseler GmbH & Co. KG, Lemgo, Germany). In addition, a vertical measuring mark was added centrally to the labial surface with the diamond disc (Komet Gebr. Brasseler GmbH & Co. KG, Lemgo, Germany).

The all-ceramic crowns were adhesively cemented. For this purpose, the ceramic bonding surfaces were treated with 5% hydrofluoric acid (Ivoclar Vivadent AG, Schaan, Liechtenstein) for 60 s. After thorough rinsing and drying by air, Monobond Plus universal primer (Ivoclar Vivadent AG, Schaan, Liechtenstein) was applied in a thin layer. After contact time of 60 s, the excess was removed by air. Subsequently, Heliobond bonding agent (Ivoclar Vivadent AG, Schaan, Liechtenstein) was applied by microbrush. To prepare the tooth stump, the dentin surface was first etched for 15 s with 35% phosphoric acid (VOCO GmbH, D-27472, Cuxhaven, Germany). After thorough rinsing and drying by air, the composite surfaces were conditioned with the Syntac (Ivoclar Vivadent AG, Schaan, Liechtenstein) dentin adhesive system. First, Syntac Primer was applied by microbrush and massaged in for 15 s, and the excess was removed by air. Second, Syntac Adhesive was applied by microbrush and thinly blown in after a reaction time of 10 s. Finally, Heliobond (Ivoclar Vivadent AG, Schaan, Liechtenstein) was applied to the tooth stump and blown into a thin layer. Adhesive cementation of the crowns was performed with Variolink II dual-curing luting composite (Ivoclar Vivadent AG, Schaan, Liechtenstein) of high viscosity. The compound, mixed over 10 s in a 1:1 ratio, was applied to the inner surfaces of the crowns so that they were one-third filled, and all walls were wetted. The restoration was initially placed under slight pressure, after which any coarse excess of luting material was removed. Final fixation of the restoration was performed under increased pressure for a few seconds. After relief, fine excess was removed by microbrushing. Subsequently, the luting composite was polymerized for 40 s per tooth surface. Further excess covering the marginal gap was removed with the aid of a curette. Finally, the oxygen inhibition layer was removed using a prophylaxis brush.

### 2.3. Margin Pallet Measurement

Investigation of the marginal gap behavior was conducted for the specimen teeth in Groups A, B, and C. For this purpose, replicas of the sample teeth were created both before and after artificial aging. These teeth were used to generate images of the entire circular marginal gap, which was subsequently measured and evaluated. To produce the replicas, the teeth were first duplicated. For this purpose, they were molded by kneadable A-silicone Flexitime Easy Putty (Heraeus Kulzer GmbH, Hanau, Germany) and thin-flowing A-silicone Dublisil 15 (Dreve Dentamid GmbH, Unna, Germany). The molding was conducted under constant vibration to ensure homogeneous distribution of the impression material without bubble formation. After 30 min, the teeth were removed from the mold by air flow. From them, Epon 812 replicas were made according to the manufacturer’s specifications.

For examination, the replicas were glued onto rotatable sample plates. A two-component epoxy resin-based adhesive, UHU plus sofortfest (UHU GmbH & Co. KG, Bühl/Baden, Germany), was used, with the shortened crown stump being used as the bonding surface. Thus, the surface to be examined could be aligned horizontally to the tabletop. To prevent electrical charge during the examination, the replicas were covered with a uniform gold layer of approximately 5 nm using a sputtering process. The images were visualized in a two-dimensional image by a LEO 1450 VP scanning electron microscope (Carl Zeiss AG, Oberkochen, Germany).

**Figure 3:**
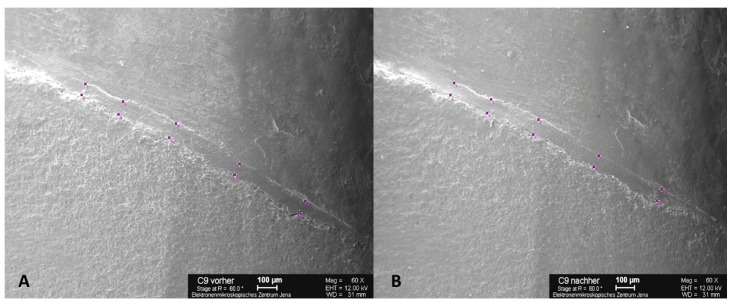
SEM images with five pairs of measuring points per section above and below the marginal gap with a minimum distance of 100 µm. Example of C9 at 60× magnification before (**A**) and after (**B**) thermocycling (TCML) for marginal gap examination. Scale bars: 100 µm.

The images were generated from as many perspectives as possible so that the electron beam was always perpendicular to the object surface. This process minimizes the distortions caused by object curvature and scattering of the electron beam. The starting point for the images was the measurement mark on the labial surface of the crowns. The tooth replicas were rotated clockwise once around their own axes, and images were obtained at 30° intervals with magnification of 60×. This process resulted in 12 individual images of each replica. Measured at the inner limit of the scale bar, the scale was 1 pixel = 2 µm. The individual images were stitched together to form a panorama using the Corel PaintShop Pro X767 software program (Version 2.0, 2013, Corel, Alludo HQ, Ottawa, IL, USA). For the marginal gap measurement, a panoramic image was used to divide the circular marginal gap into five measurement sections and the four position-dependent tooth surfaces by means of scanning electron microscopy (SEM). Starting from the labial measurement marking, the panoramic image was divided into five equally sized measurement sections, which were successively defined as “labial 1”, “mesial”, “palatal”, “distal”, and “labial 2” (clockwise for tooth 11, counterclockwise for tooth 21). The “labial 1” and “labial 2” measuring sections form the “labial” tooth surface. After TCML, the specimen teeth were de-bedded and cleaned with extreme care so that replicas could be created again for the marginal gap examination by SEM. In each case, two measuring points delimited the marginal gap—a prominent point of the outer crown margin and a point opposite to the first on the preparation margin—with their connecting line at right angles to the preparation margin so that the same measuring point could be examined before and after TCML. The marginal gap width was measured on the basis of the individual images using ImageJ software (Version 2.1, 2013, NIH, USA). On the other hand, exactly the same measuring points were selected before and after artificial aging that had to be equally represented on the respective images. A maximum of five pairs of measuring points were assigned to each individual image with a minimum spacing of 100 µm. Due to inaccuracies triggered by the object curvature, no edge measurements were performed (Figure 3). For circular evaluation of the marginal gap, 10 measurements were obtained per measuring section (20 measuring points), resulting in 50 measurements (100 measuring points) for the entire tooth. The coordinates of the pairs of measuring points were transferred to the Microsoft Excel software program (Microsoft Corporation, Redmond, WA, USA) to calculate their distance, which corresponds to the marginal gap width.

### 2.4. Implementation of Artificial Aging

Artificial aging was performed in the test laboratory of SD Mechatronik in Feldkirchen-Westerham using a CS-4.8 chewing simulator (SD Mechatronik GmbH, Feldkirchen-Westerham, Germany), combined a TC-4 thermocycler (SD Mechatronik GmbH, Feldkirchen-Westerham, Germany). For this purpose, the test specimens were embedded in Paladur autopolymerizing, colorless denture base material (Heraeus Kulzer GmbH, Hanau, Germany) up to 2 mm below the preparation margin, corresponding to the socket in the natural alveolar bone. To simulate a loading period of five years, 1.2 × 10^6^ mastication cycles were performed using a force of 50 N at a frequency of 1.3 Hz. At the same time, continuous thermocycling was performed between 5 and 55 °C with a dwell time of 30 s each, ultimately resulting in at least 10,000 thermal load cycles.

The force in the chewing simulator was vertically applied via weight plates. Due to the previously targeted embedding, the load was applied at an angle of 135° to the tooth axis. The specimen holder was positioned so that the load was centrally applied 2 mm below the cutting edge. The antagonist holder contained a steatite ball (diameter 6 mm). The correct position was checked using articulation foil. All teeth survived the TCML without fractures or loosening of the crowns, thus allowing for further test steps to be performed without any restrictions. However, some specimens showed visible micro-cracks of the all-ceramic crowns without probable gap formation. The specimen teeth were completely removed from their acrylic coatings with maximum care and cleaned so that replicas could be prepared again for the examination of the marginal gap by SEM. Additional replicas of the crowns of selected specimen teeth were created to visualize the micro-cracks via SEM.

### 2.5. Investigation of Linear Load Capacity

The linear load-bearing capacity of the specimens was investigated using the Zwick Z005 universal testing machine (Zwick GmbH & Co. KG, Ulm, Germany). For this purpose, the teeth were embedded in a specimen holder that had cylindrical holes of 10 mm in diameter. To embed the tooth in natural alveolar bone according to the socket, a wax bar was circularly placed as much as 2 mm below the preparation margin. The tooth was then positioned centrally and vertically in the hole filled with autopolymerizing resin so that the root was encased in resin up to the attached wax bar. After the resin had cured, the specimen holder was clamped in a jig angled at 45° and screwed tight. Thus, vertical force was applied at an angle of 135° to the tooth axis. The force application point was 2 mm below the incisal edge. To distribute the force evenly, thin tin foil (HELAGO—Heinz & Laufer Dentalfabrik OHG, Bonn, Germany) was inserted between the pressure die and the crown.

**Figure 4:**
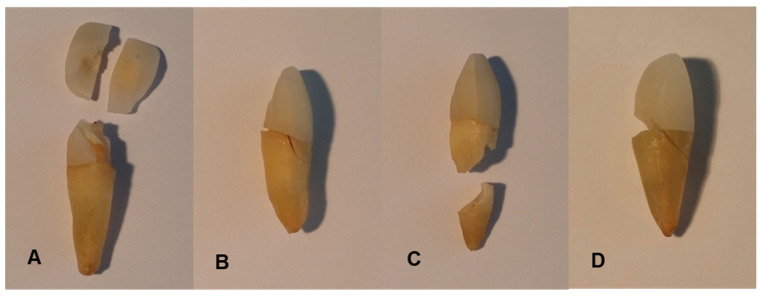
Examples of typical fracture patterns after linear loading on specimens B2 (**A**), A2 (**B**), C3 (**C**), and C8 (**D**): crown fractures (**A**), root fractures combined with marginal fissures (**B**), isolated root fractures (**C**), and complex fractures (**D**).

After applying a pre-load of 1 N, the specimen teeth were continuously loaded at a feed rate of 1 mm/min. A stress–strain diagram was recorded using testXpert 10.11 8 software (Zwick GmbH & Co. KG, Ulm, Germany). As soon as the specimens failed, the test was automatically terminated. The force at failure of the specimens corresponds to the fracture strength (Fmax). Figure 4 shows examples of typical fracture patterns, such as crown fractures (A), root fractures combined with marginal fissures (B), isolated root fractures (C), and complex fractures such as comminuted fractures with several different fracture lines throughout the entire tooth structure (D) (Figure 4).

### 2.6. Statistical Analysis and Evaluation

SPSS Statistics 22 software (IBM Deutschland GmbH, Ehningen, Germany) was used for the statistical analysis and graphical evaluation. The significance level was set at 5%. In the analysis of the linear loading capacity, a normal distribution of values was assumed for the variable of fracture strength, Fmax [N]. Comparison of the groups without TCML and those with TCML was performed by means of a one-factor analysis of variance (ANOVA). Comparison of the groups undergoing analogous treatment was performed with the aid of the *t*-test for independent samples. A total of 51 sample teeth were available (10 from Group A, 10 from Group B, 7 from Group C, 6 from Group D, 8 from Group E, and 10 from Group F). In the analysis of the circular marginal gap behavior and the marginal gap behavior in relation to the position on the tooth, a normal distribution of the values was assumed for the variability of the marginal gap width, s [µm]. Comparison of the groups before and after TCML was performed using one-way ANOVA. Comparison of the groups treated in the same way was performed with the aid of the *t*-test for connected samples. All 30 sample teeth were available before and after TCML. For graphical evaluation of the marginal gap behavior, star charts were prepared using Microsoft Excel Version 1.0 2007 software (Microsoft Corporation, Redmond, WA, USA). For this purpose, the positions of the uniformly distributed measurement points in the panorama were measured and referenced to a circle. Subsequently, the edge slit courses before and after TCML were brought into congruence.

## 3. Results

### 3.1. Analysis of the Circular Marginal Gap Behavior

The evaluation is based on the group comparison for the variable of the marginal gap width, s [µm]. The Shapiro–Wilk test showed no significant deviation of the values from the normal distribution in the respective groups. The values determined for the circular marginal gap and their analysis are shown in Table 2.

**Figure 5:**
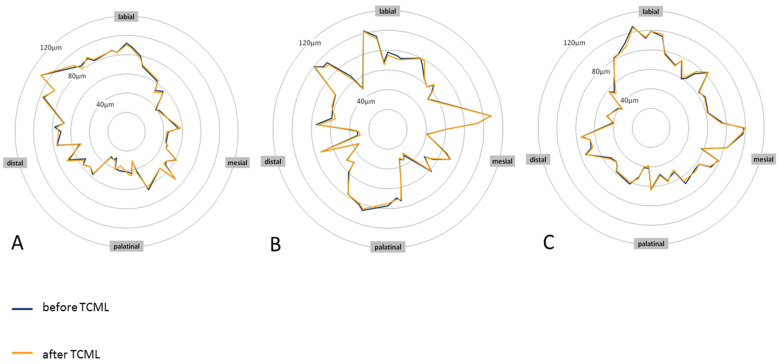
Star diagrams of specimen teeth A6 (titanium L9) (**A**), B4 (glass-fiber L9) (**B**), and C9 (glass-fiber L6) (**C**) before and after TCML showing the margin gap width.

Before TCML, it was investigated whether the differences in margin width were a result of the post system that was used. The mean values were similar, and one-way ANOVA did not show a statistically significant difference for the values of the margin width (*p* = 0.921). For the analysis of fatigue behavior associated with a change in margin width, the groups were also compared according to TCML. The mean values of the marginal gap widths barely differed between the groups. One-way ANOVA did not reveal a statistically significant difference for the values of the margin width (*p* = 0.883). Furthermore, we investigated whether there were differences in fatigue behavior depending on the respective post material or post design. For this purpose, the analog-supplied groups were compared using the paired-sample *t*-test. No clinically relevant or statistically significant changes in the marginal gap width as a result of artificial aging could be determined in any of the groups (Table 2). The graphical analysis of the marginal gap behavior using star diagrams illustrates the circular course of the marginal gap width. To show edge gap changes as a result of artificial aging, the marginal gap curves before and after TCML were brought into congruence. In Figure 5, the star diagrams of a specimen supplied with titanium L9, a specimen supplied with glass-fiber L9, and a specimen supplied with glass-fiber L6 are shown as examples (Figure 5). During the course, no relevant changes in the edge gap width resulting from artificial aging were shown at any point. This finding applies to each of the three post systems. The marginal gap widths were comparatively larger in the labial region than in other regions. The analysis further illustrates the diffuse course of the marginal gap widths in the teeth restored with all-ceramic crowns. The marginal gap widths were rarely larger than 100 µm and rarely smaller than 40 µm.

Analysis of the marginal gap behavior in relation to the position on the tooth revealed that the labial tooth surfaces had comparatively large mean values of the marginal gap width. To investigate the discrepancies, the individual tooth surfaces were compared before TCML using the *t*-tests of independent samples. For titanium L9, there was a statistically significant difference from the labial to the distal (*p* = 0.012), mesial (*p* = 0.000), and palatinal (*p* = 0.005). Similarly, for glass-fiber L9, there was a statistical significance from the labial to the distal (*p* = 0.003), mesial (*p* = 0.000), and palatinal (*p* = 0.003). Glass-fiber L6 showed a statistically significant difference from the labial to the distal (*p* = 0.001) and mesial (*p* = 0.009).

### 3.2. Linear Load Capacity Analysis

The evaluation is based on the group comparison for the variable of fracture strength, Fmax [N]. The results of the Shapiro–Wilk test did not reveal any significant deviation of the values from a normal distribution in the respective groups. Table 3 provides an overview of the determined values of the breaking strength and their analysis (Table 3).

**Figure 6:**
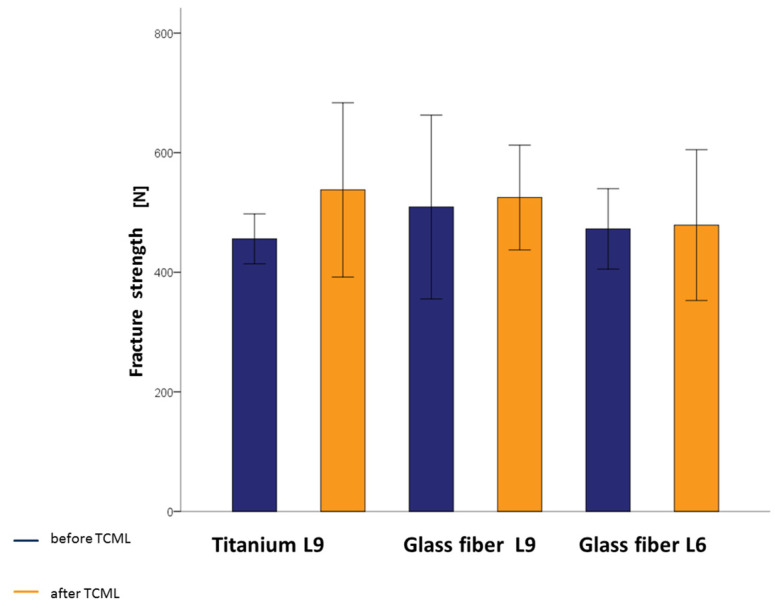
Fracture strength mean values with standard deviations of titanium L9 posts, glass-fiber L9 posts, and glass-fiber L6 posts before and after TCML.

To evaluate the influences of the post material and the post design on the fracture strength, the groups without previous TCML were compared. Slightly higher values for the teeth were found when supplied with the glass-fiber L9. This outcome was true when comparing them with both the same shape titanium L9 post and the same material in the shorter fiberglass L6 post. One-way ANOVA did not reveal any statistically significant differences for the fracture strength values (*p* = 0.596). To evaluate the influence of artificial aging on fracture strength, the groups with previous TCML were also compared. The teeth of the glass-fiber L6 group achieved slightly lower values of fracture strength, although these differences were found not to be statistically significant (*p* = 0.608) based on one-way ANOVA. In addition, the influence of artificial aging on the load-bearing capacity of the respective post material and post design was investigated. For this reason, the analog-supplied groups were compared using the *t*-test on independent samples. The groups supplied with glass-fiber L9 and glass-fiber L6 had similar mean values of fracture strength with and without TCML. The mean value of the group supplied with titanium L9 with TCML was slightly higher than that without TCML. No statistically significant correlation between fracture strength and artificial aging could be determined for any of the groups (Table 3). It is striking that higher values for fracture strength were obtained for all tested specimens after TCML. Figure 6 illustrates the fracture strength values of all investigated posts with and without TCML (Figure 6).

### 3.3. Analysis of Material Fatigue after Artificial Aging

**Figure 7:**
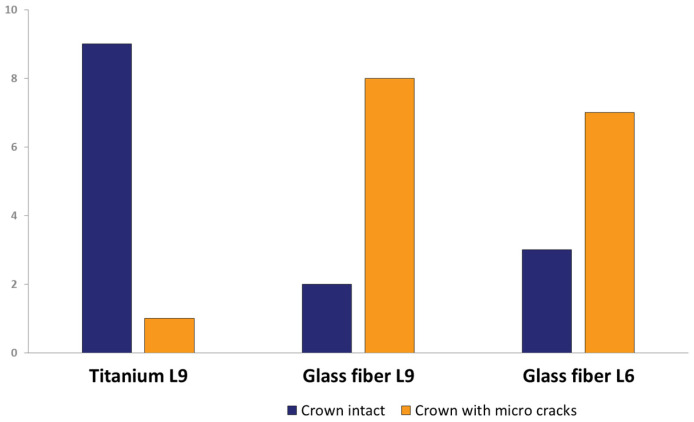
Micro-crack occurrence of titanium L9 posts, glass-fiber L9 posts, and glass-fiber L6 posts in total.

After artificial aging, 16 of the 30 specimens exhibited visible micro-cracks in the all-ceramic crowns. Neither sound-splitting fractures nor reductions in the bond strength could be detected. The position, course, and number of micro-cracks varied greatly between the affected crowns. Predominantly, the crowns of the teeth restored with glass-fiber posts were affected by micro-cracks (Figure 7). The Mann–Whitney U-test for independent samples demonstrated a statistically significant difference in the occurrence of micro-cracks between titanium L9 and glass-fiber L9 (*p* = 0.007) and between titanium L9 and glass-fiber L6 (*p* = 0.023), whereas there was no statistical significance regarding the difference in the occurrence of micro-cracks between glass-fiber L9 and glass-fiber L6 (*p* = 0.739).

**Figure 8:**
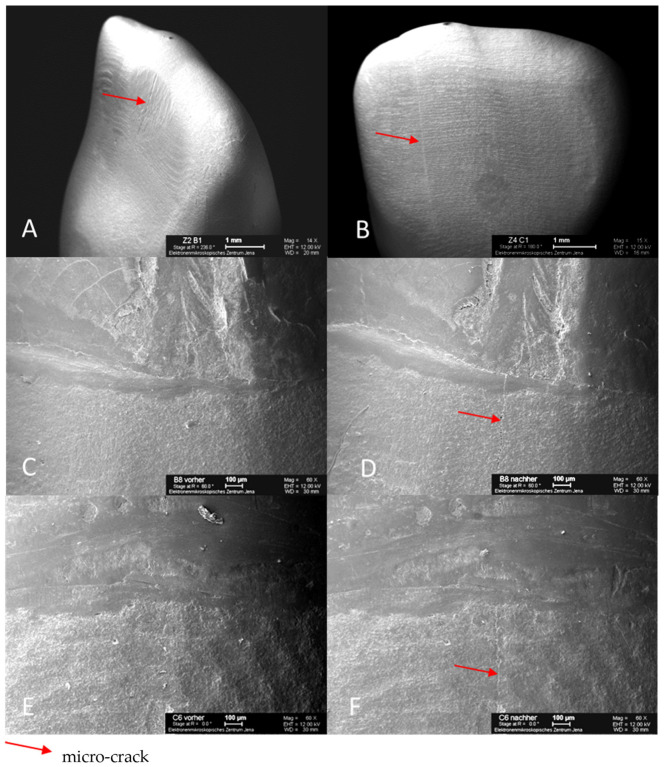
SEM images of specimen tooth B1 (glass-fiber L9) after TCML with micro-crack on the palatal surface of the crown (**A**); specimen tooth C1 (glass-fiber L6) after TCML with micro-crack on the labial surface of crown (**B**); specimen tooth B8 (glass-fiber L9) before TCML, marginal gap (**C**); specimen tooth B8 (glass-fiber L9) after TCML, with micro-crack in marginal gap area (**D**); specimen tooth C6 (glass-fiber L6) before TCML, marginal gap (**E**); specimen tooth C6 (glass-fiber L6) after TCML, with micro-crack in marginal gap area (**F**).

Representative scanning images of all-ceramic crowns with micro-cracks illustrate the dimension and the variable courses of the micro-cracks, which are marked with arrows. The same areas of the marginal gap are shown both before and after TCML. The root surface is at the top, and the all-ceramic crown is at the bottom of the SEM image (Figure 8). After TCML, fine micro-cracks can be seen in the area of the ceramic. In addition, neither widening of the marginal gap nor structural loosening is evident.

## 4. Discussion

When evaluating in vitro studies, it is of elementary importance that clinically relevant variables be considered [20,21]. Fractures of endodontically treated teeth are well-known complications in dental practice [22,23]. In view of this fact, post-endodontic restorations have primarily been analyzed their fracture resistance [24]. In contrast, the selected problem of microleakage as a risk factor for secondary lesions requires a microscopic examination of the marginal integrity [25]. With an appropriate margin definition, the evaluation of the margin width represents a reliable and comprehensible procedure for the practitioner [26]. Thus, both acute traumatic events and fatigue phenomena were examined in the present study, ensuring a comprehensive consideration of clinical fitness [24,27]. Nevertheless, it should be kept in mind that data from in vitro studies can never be directly transferred to clinical situations [6,28,29,30]. There is a need to critically correlate the results with clinical experience [30]. In vitro methods are commonly used to evaluate the loading capacity of endodontically treated teeth [31]. Due to the wide variety of test conditions and divergent methods, the obtained data are of limited comparability, and the conclusions are often contradictory. Numerous in vitro studies of teeth with inserted root posts have shown a comparable test arrangement consisting of artificial aging in terms of thermo-mechanical alternating loading, followed by linear loading to determine fracture strength [27,32,33,34,35]. In addition to a practice-oriented test setup, a complete definitive restoration of the sample tooth and the simulation of a practice-oriented treatment procedure are required to obtain clinically relevant results [6,24,28,36].

In the present study, human maxillary central incisors were used. In contrast to artificial materials, which allow for identical dimensions and constant mechanical properties, natural teeth exhibit individual variations, indicating that a larger scatter of measured values is expected [37,38]. In addition, when using natural teeth, care must be taken to ensure appropriate storage and treatment in the period after extraction so that there is no degradation of mechanical properties [37]. It is known that human maxillary incisors are often used as test specimens in in vitro studies for the evaluation of root post systems because they ensure better comparability of results [27,32,33,34,39,40,41,42]. The root posts used came from the same manufacturer, which means that the quality criteria are the same. Since the titanium L9 and glass-fiber L9 root posts have the same macro-shape of ISO size 90, reliable statements can be made about the influence of the respective materials. The glass-fiber L9 and glass-fiber L6 root posts were made from the same material and have approximately the same shaft diameter. This fact allows for valid conclusions to be drawn about the influence of the shaft length. Depending on the test conditions, the results can be compared. In addition, various investigations have been performed with the root posts of the ER system with regard to cast-on ability, surface conditioning, adhesive fixation, and analyses of mechanical and structural properties [43,44,45,46,47].

In a previous study by Schäfer (2012), shank posts made of titanium and glass fiber were investigated under the same conditions [32]. A significant decrease in fracture strength due to artificial aging was demonstrated for the teeth restored with glass-fiber posts. In particular, an increase in the palatal marginal gap was also observed in the glass-fiber post group. In our study, the average values of the fracture strength without previous TCML were 455.8 N (titanium L9), 509.2 N (glass-fiber L9), and 472.6 N (glass-fiber L6). Accordingly, the teeth restored with glass-fiber L9 exhibited the highest fracture strength, although the difference was not statistically significant. This trend is in line with the results of Schäfer (2012) [32]. Mean fracture strengths of 522.5 N for titanium and 598.8 N for glass fiber were determined for shank posts of the same material and dimensions. The comparatively lower fracture strength of the head posts investigated in the present study can be explained by greater substance removal as a result of post bed preparation [11,33,48]. The relatively high fracture strength values can be explained by a larger root cross-section of the maxillary canines [48].

In the present study, the fracture strengths with preceding TCML were 537.7 N (titanium L9), 525.0 N (glass-fiber L9), and 478.9 N (glass-fiber L6), on average. Due to different test setups and variable test parameters, the comparability of resilience studies is difficult. In this respect, it is not surprising that studies can produce contradictory results. In view of this fact, fracture strength values should always be seen in the context of the chosen study design [2,26,49,50]. The fracture strength of healthy teeth serves as the basis for assessing fracture strength values of differently treated teeth. Roberto et al. (2012) determined average fracture strength values of 750 N for healthy maxillary central incisors, which would decrease to an average of 670 N after endodontic treatment [51]. Since root posts do not exert a reinforcing effect on endodontically treated teeth, contrary to earlier views, a further decrease in fracture strength as a result of post bed preparation can be expected [12,52,53,54]. On the basis of the present study, no statements can be made about reinforcing or weakening influences because teeth with inserted root posts were examined without exception. In this respect, the teeth restored with glass-fiber L6 showed slightly lower fracture strength, although no statistically significant difference could be demonstrated. This finding is in line with the conclusions of recent studies according to which the post length of adhesively placed root canal posts had little influence on the fracture resistance of endodontically treated teeth. Consequently, it is necessary to reconsider the dogmas regarding the minimum post length that have been repeated for decades [54,55]. The study by Shiavetti et al. (2009) also showed that there is no significant difference in fracture resistance between the lengths of glass-fiber root posts. Adhesively placed glass-fiber root posts with lengths of 5 mm, 7 mm, and 8 mm were investigated for fracture strength [56]. For completeness, however, it must be mentioned that there are other root-post materials. Stainless-steel root-posts were investigated for fracture resistance by Amarnath et al. (2015) in comparison with glass-fiber root-posts [57]. In their study, the fracture resistance of stainless steel was significantly higher than that of glass fiber [57]. Compared to the values without TCML, no reduced fracture strength as a consequence of artificial aging was observed in any of the post groups. The obtained results document a positive influence of the head post design on the fatigue resistance of the material. It was notable that the mean value of titanium L9 with TCML was slightly higher than that without TCML, but this difference was not statistically significant. This phenomenon is not unique and can be explained by the use of natural sample teeth [31,58,59]. Due to the different dimensions and individual mechanical properties of human teeth, measured values can show a large scatter [37,38,39,60]. According to TCML, the mean fracture strengths of 433.3 N for the titanium group and 270.5 N for the glass-fiber group were determined. The advantageous fatigue resistance of head posts is attributed to the greater stability and flexural strength in the cervical region, as well as more favorable force distribution within the tooth [9,61,62,63]. Despite slightly lower initial fracture strength, the investigated head posts did not show any decrease in fracture strength as a result of TCML, seemingly striking a good balance [52]. The fracture strength values predicted in the pre-determination exceeded the maximum expected bite force values regardless of the post material, post length, or artificial aging [64,65,66,67,68].

In the present study, the mean marginal gap widths after cementation of the crowns were 66.10 µm (titanium L9), 65.71 µm (glass-fiber L9), and 66.66 µm (glass-fiber L6). Accordingly, there was no statistically significant difference among the groups. In addition, the values corresponded to the glass-fiber post group from the study by Schäfer (2012), which had an average edge gap width of 63.5 µm [32]. This finding indicates a comparable test setup and reproducible treatment processes. In the titanium post group from the study by Schäfer (2012), significantly greater marginal gap widths of 111.4 µm on average were found, which was explained by inaccuracies in the manufacturing process or during measurement of the specimens, as well as the influence of the training effect [32]. The marginal gap widths around a mean value of 66 µm and with individual values rarely greater than 100 µm and smaller than 40 µm represent highly acceptable treatment results. A systematic research study by Contrepois et al. (2013) revealed that 94.9% of the margin gap values determined were less than or equal to 120 µm, with the largest margin gap being 174 µm and the smallest margin gap being 3.7 µm [69]. In the literature, margin widths of 50 µm to 100 µm are considered clinically desirable, and values of up to 200 µm are still tolerable [70,71,72,73,74]. While Dreyer-Jørgensen (1958) demanded values less than 50 µm, marginal gap widths of 70 µm are today considered clinically optimal [75,76,77,78] and 110 µm clinically realistic [75,76,77,78]. Under optimum conditions, Bindl et al. (1999) achieved mean marginal gap widths of 59.9 µm for CEREC-fabricated anterior crowns [79,80].

After TCML, the marginal gap widths in the present study averaged 66.10 µm (titanium L9), 65.68 µm (glass-fiber L9), and 66.83 µm (glass-fiber L6). Consequently, no statistically significant differences could be found either among the groups or when compared to the values before TCML. Within the experimental setup, all three pin variants tested showed high resistance to marginal microcracking due to the design of the post head, which provides high resistance to oblique masticatory forces. However, 16 of the 30 sample teeth showed visible micro-cracks in the upper regions of the all-ceramic crowns, with one exception involving only glass-fiber post restorations. There were no detectable cracks or fractures, nor was there any reduction in bond strength. Although the defects were visible, all of the specimen teeth survived the TCML. In addition, the affected specimen teeth exhibited high fracture strength and unaltered marginal integrity under the selected examination method. Under long-term loading, the development of micro-cracks is a characteristic failure pattern of all-ceramic restorations, which speaks to the clinical relevance of the experimental setup [32,81,82,83]. It is also known in the literature that the formation of micro-cracks depends on the type of ceramic and that the bond strength is related to the surface coating of the root post system [84]. A special feature of this study is that the location of the marginal gap formation was also analyzed. For titanium L9, there was a statistically significant difference from the labial to the distal (*p* = 0.012), mesial (*p* = 0.000), and palatal (*p* = 0.005). Similarly, for glass-fiber L9, there was a statistical significance from the labial to the distal (*p* = 0.003), mesial (*p* < 0.000), and palatal (*p* = 0.003). Glass-fiber L6 showed a statistically significant difference from the labial to the distal (*p* = 0.001) and mesial (*p* = 0.009). No obvious reason for the differences was found. It is conceivable that the natural tooth shape of the teeth used was different from the shape of the crown restoration and that this shape did not correspond to the natural anatomy.

A limitation of this study was in reproducing a complex in vitro system in vivo. It is similarly difficult to simulate a force system similar to the human masticatory apparatus in vivo in terms of artificial aging of the crowned teeth with root posts. However, clinically proven and commonly used materials, as well as human teeth, were used to render the experimental system as realistic as possible. Moreover, force application on the restoration was investigated at the same time as artificial aging, and in its entirety, the process is very similar to the natural clinical process.

## 5. Conclusions

The present in vitro study showed that the root post material and the root post length had no significant influence on the fracture strength of the test teeth before and after artificial aging. Furthermore, we found that, as described in the literature, micro-cracks in the area of the restoration and the marginal gap can occur as a result of material fatigue. However, artificial aging has no influence on the marginal quality as a function of the root post material and the root post length. Only the marginal gap location is affected.

As demonstrated in this study, the long-established Erlanger post system combines well with prosthetic crown restoration using the newer CEREC system. It is often assumed that metal posts have lower fracture strength than glass-fiber posts. This assumption could not be demonstrated in this extensive test model. Hence, both titanium and glass-fiber root posts proved equally effective in combination with CEREC ceramic crowns. Based on the determined fracture strength values and investigated marginal gap behaviors, all three root post variants are recommended for clinical application.

The visible micro-cracks in the all-ceramic crowns that occurred as a result of artificial aging, which almost exclusively affected the glass-fiber post restorations, indicate the need for further investigations to determine the causes of micro-cracks and to help develop prevention strategies.

## Figures and Tables

**Table 1:** Manufacturer’s specifications for the used root posts in material and form: titanium L9, glass-fiber L9 and glass-fiber L6.

		Titanium L9	Glass-Fiber L9	Glass-Fiber L6
Designation		ER Kopfstift	ER DentinPost X	ER DentinPost X
		49L9.000.090	445L9.000.090	444L6.000.070
**Material**		Pure titanium	Glass-fiber-reinforced composite	
Composition		≤0.35% O	≈60% glass fiber	
		≤0.30% Fe	≈40% epoxy resin	
		≤0.06% C		
		≤0.05% N		
		≤0.013% H		
		≤0.04% other		
**Form**		Head post with conical shank		
Pitch angle		2.1°		
Head	Length	4.5 mm		
	Ø coronal	2.0 mm		
	Ø cervical	2.8 mm		
Shaft	Length	9.0 mm		6.0 mm
	Ø cervical	1.56 mm		1.58 mm
	Ø apical	0.90 mm		1.14 mm

**Table 2:** Overall view of the circular marginal gap width, s [µm], before and after TCML of titanium L9 posts, glass-fiber L9 posts, and glass-fiber L6 posts.

			Mean	Standard Deviation	Significance *p* ≤ 0.05	Significance between the Groups before TCML	Significance between the Groups after TCML
Titanium L9	before TCML	N [10]	66.10	6.38	0.996		
	after TCML	N [10]	66.10	6.27			
Glass-fiber L9	before TCML	N [10]	65.71	5.39	0.815	0.921	0.883
	after TCML	N [10]	65.68	5.48			
Glass-fiber L6	before TCML	N [10]	66.66	3.61	0.141		
	after TCML	N [10]	66.83	3.50			

**Table 3:** General overview of the fracture strength Fmax [N] before and after TCML of titanium L9 posts, glass-fiber L9 posts and glass-fiber L6 posts.

			Mean	Standard Deviation	Significance *p* ≤ 0.05	Significance between the Groups before TCML	Significance between the Groups after TCML
Titanium L9	before TCML	N [6]	455.8	41.7	0.123		
	after TCML	N [10]	537.7	145.7			
Glass-fiber L9	before TCML	N [8]	509.2	153.8	0.801	0.596	0.608
	after TCML	N [10]	525.0	87.8			
Glass-fiber L6	before TCML	N [10]	472.6	67.3	0.894		
	after TCML	N [7]	478.9	126.1			
